# Forward from the Editor-in-Chief

**DOI:** 10.1136/fmch-2024-002868

**Published:** 2024-04-12

**Authors:** Li Li

**Affiliations:** 1 Department of Family Medicine, University of Virginia School of Medicine, Charlottesville, Virginia, USA; 2 UVA Comprehensive Cancer Center, University of Virginia, Charlottesville, Virginia, USA

Family medicine in the USA, born out of the timeless and universally needed role of the general practitioner, has enjoyed tremendous growth since its founding in 1969. It is now one of the largest medical specialties with an active workforce of approximately 118 000 board-certified family physicians. Many of the 165 Departments of Family Medicine at US academic institutions celebrated their 50th anniversary in the past few years. There are currently over 750 residency programmes in the USA and this number continues to grow, driven by the widespread shortage and inequitable geographical distribution of primary care physicians nationwide.

Family medicine, a generalist specialty that is relationship based and broadly encompassing, plays a fundamental role in healthcare systems and delivery of high-quality primary care. The National Academies of Sciences, Engineering and Medicine’s 2021 consensus report, Implementing High-Quality Primary Care, reinforced its 1996 report, Primary Care: America’s Health in a New Era, finding that primary care is ‘a common good’ and ‘the only heath care component where an increased supply is associated with better population health and more equitable outcomes’.^1^ ^2^ Despite this widely recognised indispensable role of family medicine, family physicians in the USA and much of the world have been increasingly experiencing a professional identity crisis: their scope of practice is narrowing, the pipeline of workforce is shrinking, federal funding of primary care research has been stagnant, and erosion of practice by corporate transactional medicine is rampant.

This special series in Family Medicine and Community Health, entitled ‘Storylines of Family Medicine,’ is a timely reflection of the past, present and future of family medicine. The 99 short essays authored by 136 front-line family physicians, educators and scholars share profound wisdom and insight into what defines and differentiates family medicine. These rich articles go beyond Starfield’s 4Cs of comprehensiveness, first contact, coordination and continuity, to bring to life family medicine’s integrating, personalising focus on the whole person in their family and community context.^3^ The ‘Storylines of Family Medicine’ series is a powerful reminder that family medicine needs to be nimble as it embraces difficult challenges and innovative opportunities while staying true and firm to these foundational core values and to our commitment to ‘Health for All.’

Although Storylines of Family Medicine is largely targeted towards a US audience, I have no doubt it will resonate with readers from around the world. It is also my hope that these freely accessible essays will be widely used to train and develop future generation of physicians who embrace the core values of primary care as a common good!
